# An Electrochemical Approach to Follow and Evaluate the Kinetic Catalysis of Ricin on hsDNA

**DOI:** 10.3390/life11050405

**Published:** 2021-04-29

**Authors:** George Oliveira, José Maurício Schneedorf

**Affiliations:** Department of Biochemistry, Institute of Biomedical Sciences, Federal University of Alfenas, Alfenas 37130-000, Brazil; jose.dasilva@unifal-mg.edu.br

**Keywords:** ricin, square wave voltammetry, kinetic analysis, hsDNA

## Abstract

International authorities classify the ricin toxin, present in castor seeds, as a potential agent for use in bioterrorism. Therefore, the detection, identification, and characterization of ricin are considered the first actions for its risk assessment during a suspected exposure, parallel to the development of therapeutic and medical countermeasures. In this study, we report the kinetic analysis of electro-oxidation of adenine released from hsDNA by the catalytic action of ricin by square wave voltammetry. The results suggest that ricin-mediated adenine release exhibited an unusual kinetic profile, with a progress curve controlled by the accumulation of the product and the values of the kinetic constants of 46.6 µM for *Km* and 2000 min^−1^ for *kcat*, leading to a catalytic efficiency of 7.1 × 10^5^ s^−1^ M^−1^.

## 1. Introduction

Ricin, found in the endosperm of castor seeds (Ricinus communis L.), is considered one of the most potent toxins of plant origin, with a lethal LD_50_ of only 5 µg/kg in mice (inhalation), with the symptoms appearing up to 4 h after inhalation and the irreversibility of the lethal condition between 6 to 12 h [1,2]. Its action mechanism involves the inactivation of protein synthesis by an *N*-glycosilase activity towards a specific adenine residue in ribosomal DNA. Ricin has gained recent attention from governments and the international scientific community due to its potential use in bioterrorism, the low cost for plant cultivation, the relative simplicity for extraction, and a good stability of the phytotoxin [3,4]. In this context, ricin is classified by the US Centers for Disease Control and Prevention (CDC, Atlanta, GA, United States) as a category B bioterrorism agent [5,6]. The structure of ricin comprises a heterodimer of approximately 65 kDa composed by a catalytic subunit (RTA, 267 amino acids residues, 32 kDa) joined to a binding subunit (RTB, 262 amino acids residues, 33 kDa) by a disulfide bond between Cys259 of RTA and Cys4 of RTB [7]. RTB binds to portions of galactose residues on the eukaryotic cell surfaces, favoring the cell entry and following a retrograde transport from the Golgi complex to the endoplasmic reticulum, where the phytotoxic action of ricin manifests itself [8]. The RTA subunit acts as a ribosome inactivator type II (RIP), causing cell death by the depurination of the adenine 4324 and leading to protein synthesis inhibition [9].

Countermeasures for ricin containment include significant investments in vaccine development, the synthesis of protein cell traffic inhibitors, the rational synthesis of competitive inhibitors for RTA or bind blockers for RTB [10], and improved methods for the identification and characterization of the phytotoxin. The early detection of the active toxin may contribute to an appropriate risk assessment during suspected exposure and the development of therapeutic and medical countermeasures [5].

Although ricin is classified as a ribosome *N*-glycosilase due to the depurination of the specific adenine 4324 located in the 28S rRNA SRL, the phytotoxin can also be described as a polynucleotide:adenosine *N*-glycosylase, because of the depurination of alternative adenosine-containing RNA and DNA substrates [4,5]. Thus, monitoring the adenine released from the catalytic action of ricin is essential to establish the catalytic mechanism of the toxin, and facilitate the search for new inhibitors against the lethal action of the toxin. The action of its catalytic portion (RTA) is often reported against intact ribosomes, DNA and RNA substrates, natural or synthetic, and also on synthetic short-chain oligonucleotides with a base-paired stem and a GAGA tetraloop [11]. In this work, we have used herring sperm DNA (hsDNA) as the substrate for ricin activity.

Herring sperm DNA (hsDNA) is considered as a target molecule in studies of antitumor and anticancer drugs, binding agents, the modulation of DNA structure and function [12], and physicochemical behavior in solution [13,14]. As reported elsewhere, hsDNA can be considered as a good substrate for ricin [15,16]. In our experiments, we have used hsDNA because of its easiness for acquisition and chemical stability.

Methods used to characterize the ricin activity from adenine release include the detection of the purine by molecular absorption [16], luminescent assay [17] and high-pressure liquid chromatography (HPLC), with or without derivatization of the sample [11,15,18].

Here, we report the electrochemical technique of square wave voltammetry (SWV) to follow the enzymatic activity of ricin and the resulting kinetic analysis. The method is focused on the real-time traces of adenine released from herring sperm hsDNA by oxidation of the analyte on the surface of an unmodified carbon paste electrode. This electroanalysis technique has some advantages for the catalytic characterization of ricin, such as low cost, rapid response, relative simplicity for detection setup, high sensitivity [19], and the possibility to use small and portable devices with few microliters on a surface of a disposable solid phase electrode (SPE). Furthermore, to the authors’ best knowledge, this is the first time that the ricin activity could be monitored on double-stranded nucleic acid as a substrate together with unmodified surface electrodes, with both characteristics able to be easily implemented in portable electrochemical devices as a point-and-care detection of its harmful activity.

## 2. Materials and Methods

### 2.1. Ricin Purification

Ricin was prepared as described by Ngo et al. [20]. Briefly, ground castor bean seeds were delipidated with concentrated hexane:acetone solution (1:1). The samples were homogenized in a 0.15 M NaCl solution and allowed to sit for 24 h at 4 °C. The crude extract was filtered in Whatman paper no. 2, and the pooled filtrate was adjusted to 70% ammonium sulfate saturation and stirred for 4 h at 4 °C. The solution was then centrifuged for 10 min (17,900× *g* at 4 °C) and the precipitate dissolved in distilled water, and extensively dialyzed for 72 h. The crude ricin extract was purified with a Sephadex G-100 column following equilibration with the saline solution, and the eluate was monitored at 280 nm (Libra S22 UV/Vis, Biochrom LKB, St. Albans, UK).

To check the purity of the eluated, the samples corresponding to the spectrophotometric peaks were run on 12% SDS-PAGE under reduced conditions according to the method described by Laemmli (1970) [21]. The protein content was determined by Bradford assay in the purified samples [22].

Aiming to test the potency of the purified ricin, cell viability was conducted with HEP2 cells by the MTT assay [23]. The samples (10,000 per well) were seeded in 96-well plates 24 h prior to the assay, and the cells were treated with varying ricin concentrations for 24 h before the test. Cell viability was defined as the percentage of surviving cells obtained relative to the cells treated with DMSO as a control [24]. The IC_50_ value (inhibitory concentration at 50% inhibition of cell growth) was calculated from the adjustment of a logarithmic equation to the data by using R (R Core Team, 2018, Vienna, Austria).

For the assay of specific enzymatic activity, aliquots of the crude extract and purification steps were incubated with 5 µM of the denatured hsDNA solubilized in 50 mM sodium acetate buffer, pH 4.6, under agitation for 60 min at 25 °C. The adenine released during ricin purification activity in hsDNA was analyzed by the electrochemical assay described below.

### 2.2. Electrochemical Assays

All experiments were conducted with a PG39MCSV potentiostat–galvanostat (Omni Metra Instrumentos Científicos Ltda, Nova Friburgo, Brazil), using a conventional three-electrode system in a 10 mL jacketed cell coupled to a water circulation bath (Q214-SC, Quimis, Diadema, Brazil) at 25 °C, and at a 0.01 °C resolution. The working electrode was an unmodified carbon paste electrode (CPE) built in the laboratory, with a geometric area of 4.91 mm^2^. A platinum wire was used as an auxiliary electrode and Ag/AgCl with saturated KCl as the reference electrode. The CPE electrode was prepared by mixing graphite powder and mineral oil at a mass ratio of 75/25 (% *w*/*w*). The carbon paste was compacted in the hole of a glass tube with a conductive wire for contact with the potentiostat. The surface of carbon paste electrodes was polished over vegetal paper to ensure a smooth surface.

Adenine released during the depurination activity of holoricin on hsDNA was measured by square wave voltammetry (SWV) with the following experimental conditions: 50 Hz of frequency; a potential window from +0.5 to +1.4 V; sweep increment (∆Es) of 5 mV, and amplitude of 50 mV. Ricin was added to the electrochemical cell containing the supporting electrolyte (acetate buffer, pH 4.6) under continuous stirring and a final concentration of 1.5 nM. After a potentiometric stabilization period of 30 min, hsDNA previously denatured was added at varying concentrations (2.5 to 50 µM), and at 10-min intervals between readings. The denatured hsDNA was obtained following Bevilacqua et al. [15]. Briefly, native hsDNA was dissolved in acetate buffer (pH 4.6), heated in a water bath at 95 °C for 5 min, followed by cooling on ice for another 5 min. The adenine released was previously standardized for the analytical calibration by successive additions of an adenine stock solution in an hsDNA sample using a micropipette. After each addition, the solution was stirred to homogenize its composition before data acquisition by SWV. 

For measurements of product inhibition, 0–80 µM adenina were added to the activity assays. All the experiments were conducted in triplicate.

### 2.3. Measurement of Released Adenine by HPLC

The enzymatic catalysis of ricin was also determined by the proposed HPLC method as a comparison [25]. The liquid chromatography (HPLC) profiles were obtained using Shimadzu Prominence equipment (Tokyo, Japan) model LC-100 with automatic injector (40 µL) and a UV/VIS detector at 280 nm. The system was equipped with a C18 analytical column (Shimadzu CLC-ODS, 250 mm × 4.4 mm internal diameter × 5 µm). Chromatographic analyzes were performed in the isocratic mode following the methodology proposed by Hines et al., changing the detection system to UV/VIS at 280 nm [25]. The mobile phase consisted of a 0.1% formic acid solution, at a flow rate of 1.0 mL min^−1^ adjusted with a high-pressure pump. The execution time of the method was 10 min, and all experiments were performed at 25 °C. For every 10 injections, the column was purged with 70% acetonitrile to remove ricin and retained substrate. 

For the activity assay, 5 µM of the denatured hsDNA solubilized in 50 mM sodium acetate buffer, pH 4.6, was incubated with ricin (1.5 nM) under agitation and a volume total reaction of 3 mL at 25 °C. At regular intervals, a 100 µL aliquot was removed from the reaction system and added to an HPLC sample vial containing a mixture of 10 µM of allopurinol (internal standard) in 0.1% formic acid. They were then injected into the HPLC for reading.

## 3. Results and Discussion

### 3.1. Ricin

The presence of purified ricin was observed in two main fractions under reduced SDS-PAGE conditions (Figure S1A, Supplementary Material). Purification efficiency was estimated by determining the enzyme activity yield and the purification factor [26]. From the results of the enzymatic activity and protein concentration, the specific activity, purification factor, and yield were determined. Using this purification procedure, the total activity decreased as the purification process took place, as expected, while the specific activity was increased by one hundred and fourteen times up to 0.28 µM min^−1^ µg^−1^ (Table S1). The cytotoxic effect of the purified ricin on HEP2 cells revealed an IC_50_ value around 41.2 ng/mL, thus suggesting that the samples could affect the viability of the cells in a dose-dependent manner (Figure S1B, Supplementary Material) [20,27].

### 3.2. Enzymatic Catalysis of Ricin

**Figure 1:**
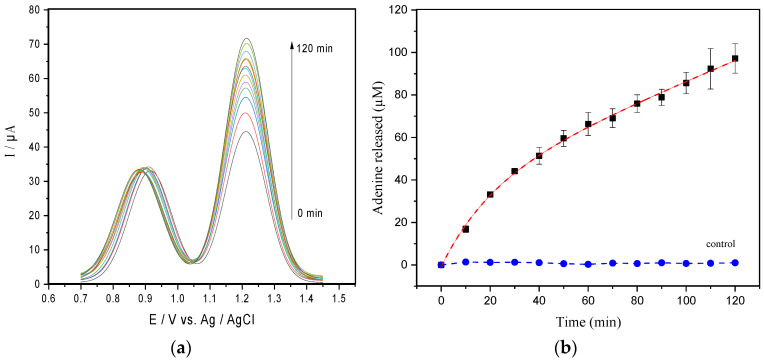
(**a**) SWV readings of the adenine released from 50 µM of hsDNA catalyzed by 1.5 nM ricin at CPE in the acetate buffer pH 4.6. (**b**) Progress curve obtained from current peaks of the SWV data (the dashed line represents the best exponential fit to the data). The control curve represents the assay carried out in absence of 50 µM of hsDNA.

SWV followed the adenine release obtained from the catalytic activity of 1.5 nM of ricin on 50 µM of hsDNA, and the data were recorded for 90 min at 10-min intervals (Figure 1a). A linear correlation was observed in a range from 0 to 95 µM of adenine, and the calibration curve was established as ∆Ip (µA) = 0.283 ± 0.004 C_adenine_ (µM) + 0.135 ± 0.047 (R2 = 0.998), and with the detection limit (LOD) estimated to 0.22 µM (S/N = 3, Figure S2, Supplementary Material).

The progress curve shown in Figure 1b suggests that ricin-mediated adenine release exhibited a fast initial phase (burst phase) followed by a decay of product release rates during the slowdown. 

Aiming to evaluate the kinetic regime obtained with the proposed strategy with another approach, the determination of the released adenine was recorded for 120 min by HPLC. The unusual shape of the curves found for hsDNA catalysis by ricin from SWV was also confirmed by that approach (Figure S3, Supplementary Material). Although this unusual shape of the progress curves has been reported previously for DNA depurination by ricin [15,16,25], no kinetic characterization was reported at the authors’ best knowledge.

Aiming to understand the kinetic mechanism of the ricin on hsDNA, the most likely explanations for the unusual shape of the curves were investigated. First, we certified that the enzyme reaction was not affected by phenomena such as spontaneous hydrolysis or hsDNA renaturation. Thus, a sample containing only hsDNA in the absence of ricin (control, Figure 1b), suggests that possible hsDNA restructuring phenomena do not significantly interfere in the results. We also considered the partial inactivation of the enzyme caused by the time-dependent conformational change of the enzyme structure; as an example, the dilution in the reaction mixture, protein–protein association phenomena, or something similar and by the substrate binding [28,29]. However, the inactivation of the enzyme was ruled out, as demonstrated by Selwyn’s test (Figure S4, Supplementary Material) [30].

Then, we considered the inhibition by product, i.e., adenine released from the ricin activity, so that, in initial times, the rate of product formation corresponds to the uninhibited rate of the enzymatic reaction. After an increase in the concentration of the product formed by the reaction, however, the rate changes due to a lowest catalysis rate [28]. To study the product inhibition, we used the approach proposed by Cao and De La Cruz (2013) to obtain the kinetic parameters of the steady-state enzyme from non-linear kinetic time courses with product inhibition and/or substrate depletion [31]. The following equation (1) can be fitted to the data to analyze the unusual progress curve of the kinetics ricin in hsDNA.

(1) $$\left[P\right]=\frac{v_{ss}}{k_{obs}}\left(1-e^{-k_{obs}t}\right)$$

where [*P*] is product concentration at time *t*, and the limiting forms of the enzyme have kinetic parameters: *v_ss_*, the initial rate enzyme cycling (e.g., no product inhibition or substrate depletion); and *k_obs_*, a term describing the reduction in cycling velocity that causes non-linearity in time courses, respectively.

**Figure 2:**
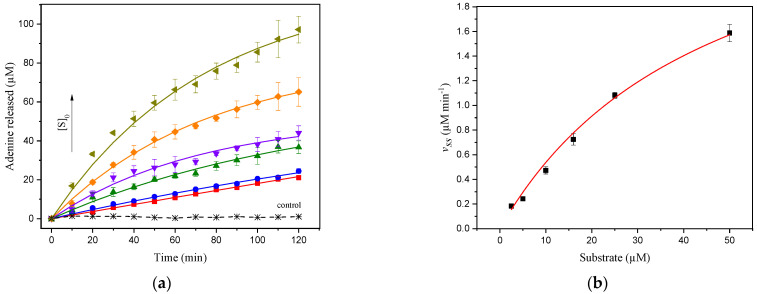
(**a**) Progress curves from SWV of adenine release of 2.5, 5.0, 10, 16, 25, and 50 µM from hsDNA catalyzed by ricin at 1.5 nM at CPE in acetate buffer (pH 4.6). The solid curve represents the fit of Equation (1) to the experimental data. The control curve represents the assay carried out in the absence of 50 µM of hsDNA; (**b**) dependence of the initial velocity (*v_ss_*) on the hsDNA concentration. The solid curve represents the kinetic curve fitted of the Michaelis–Menten equation for ricin catalysis on the hsDNA substrate.

The depurination reaction concomitant to the catalytic action of ricin was monitored for hsDNA concentrations ranging from 2.5 to 50 µM (Figure 2a), and with the overall reaction dependent on the substrate concentration. The dependence of the initial velocities obtained from fitting data to Equation (1) (*v_ss_*) on hsDNA concentration follows a regular hyperbolic saturation form (Figure 2b), and fitting to the Michaelis–Menten equation produced estimates of the Michaelis–Menten constant (*Km*) of 46.6 ± 16.5 µM and maximum velocity (*Vmax*) of 3.0 ± 0.8 µM min^−1^. Taking into account the catalytic rate constant (*kcat*) value of 2000 min^−1^ as determined from *kcat* = *Vmax*/[E] in the steady-state phase, where [E] represents the molar concentration of total enzyme, the specificity constant for ricin on hsDNA, *kcat*/*Km*, was calculated as 7.1 × 10^5^ s^−1^ M^−1^.

Although the authors could find neither *kcat* nor *Km* values for the catalytic activity of ricin on hsDNA as a substrate in the literature, some parameters were attained with synthetic substrates or ribosomes under steady-state catalysis conditions [11,17,18,32]. In this sense, the *kcat/Km* value found in this work was reported in the same order of magnitude by Chen et al. [18] (4.5 × 10^5^ M^−1^ s^−1^) for ricin A-chain subunit activity with 14-base stem-loop RNA substrate, and close to that reported by Aitbakieva et al. (3.8 × 10^6^ M^−1^ s^−1^) [32] when using 28S rRNA substrate. In contrast, Tan et al. [11] when using synthetic oligoribonucleotides and as by Sturm and Schramm [17] when using rabbit reticulocyte ribosomes or yeast ribosomes as substrate, reported higher *kcat/Km* values of 1.1 × 10^7^ M^−1^ s^−1^ and 2.6 × 10^8^ M^−1^ s^−1^, respectively. These reported results revealed a wide range for the kinetic parameter values obtained with different substrates, and could be due to different assay conditions, methods of detection, oligoribonucleotides and kinetic models used.

**Figure 3:**
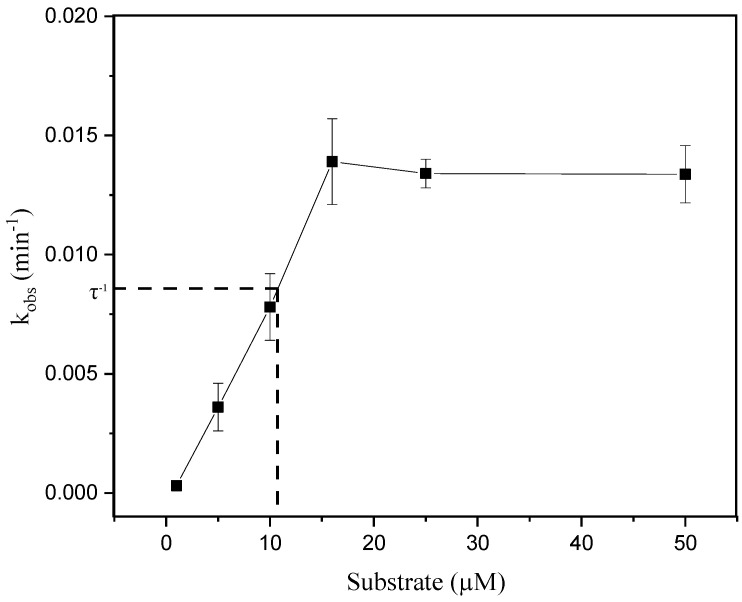
Dependence of *k_obs_* values with substrate concentrations. The solid line through the data points is for visualization only. The dashed line indicates the value of the reciprocal of the data acquisition time interval (τ^−1^).

To confirm product inhibition taking place during hsDNA depurination, a diagnosis based on the variation of the value of *k_obs_* with the concentration of substrate was followed (Equation (1)). Changes in *k_obs_* with increasing hsDNA concentrations are shown in Figure 3. According to the data, the values of *k_obs_* < τ^−1^, where τ represents the time range of data acquisition, increase with the concentration of substrate, indicating a regime in which the inhibition of the product predominates [31]. However, values of *k_obs_* > τ^−1^, which tend to plateau with substrate increasing, indicate that the inhibition of the product and the depletion of the substrate contribute in a similar way to the degree of non-linearity observed in the kinetics of the enzymatic cycle.

**Figure 4:**
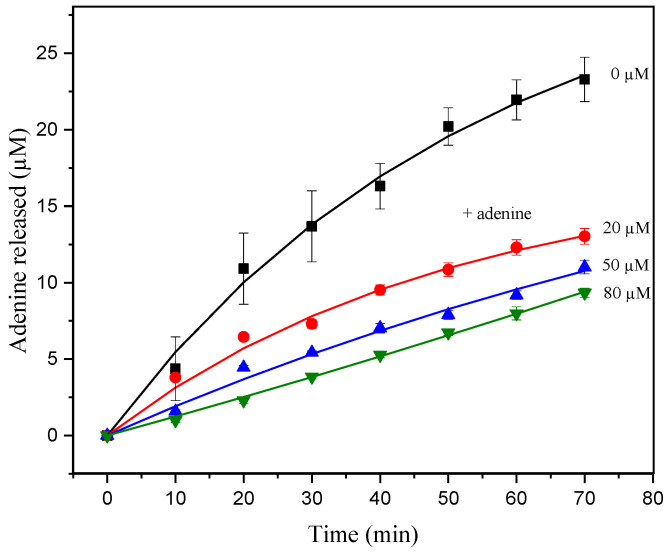
Progress curve of product release from hsDNA 10 µM catalyzed by ricin at 1.5 nM, with adenine concentrations of 0, 20, 50, and 80 µM at CPE in acetate buffer (pH 4.6). The solid curve represents the fit of Equation (1) to the experimental data.

Additionally, to certify the product inhibition hypothesis during hsDNA depurination, a classical inhibition assay was performed. Increasing amounts of adenine were added during the ricin activity assays by fixing the hsDNA concentration (Figure 4) [33]. The data presented in this study show that the addition of adenine results in a significant decrease in the amount of product formed (Figure 4).

Thus, the value for inhibitor concentration giving 50% inhibition (*IC*_50_) can be attained from these data, following Equation (2) [28].

(2) $$\frac{v_{ss}}{v_{0}}=\frac{1}{1+\frac{\left[I\right]}{IC_{50}}}$$

where *v_ss_* is the initial velocity in the presence of inhibitor at concentration [*I*], and *v_0_* is the initial velocity in the absence of inhibitor. The *IC*_50_ evaluated from initial velocity data in the presence of adenine exhibited an apparent *IC*_50_ value of 24.8 ± 1.6 µM. The apparent *IC*_50_ values determined from these experiments suggest that inhibition by adenine has a significant effect during in vitro assays (Figure 2a). Thus, the progress curves seem to correspond to the product inhibition phenomenon, in which, after a few turnovers, the concentration of the product formed in the reaction is high enough to partially inhibit ricin.

## 4. Conclusions

In this work, we have used square wave voltammetry to monitor the catalytic activity of ricin on hsDNA, following the determination of kinetic parameters. Our findings revealed that the ricin catalysis can be determined amperometrically. However, the adenine released from the catalytic activity of ricin on hsDNA is strictly controlled by the accumulation of the product, which results in an unusual kinetic profile for the progressive curves. The understanding of the kinetic mechanism of the catalysis of hsDNA by ricin may facilitate the rapid detection of the active toxin by electrochemical devices, together with the search for new inhibitors against its lethal action. 

## Data Availability

Not applicable.

## Supplementary Materials

The following are available online at https://www.mdpi.com/article/10.3390/life11050405/s1, Figure S1: purification and characterization of ricin, (A) SDS-PAGE (12%) of samples under reduced conditions, (B) effect of a pooled fraction of purified ricin on the viability of HEP2 cells for *IC*_50_ determination, Figure S2: the analytical curve of adenine obtained by successive additions of standard adenine in a range from 0 to 95 µM in a solution containing hsDNA at 16 µM, Figure S3: progress curve of adenine release from hsDNA (5 µM) catalyzed by ricin at 1.5 nM as obtained by HPLC, Figure S4: Selwyn’s test (enzyme inactivation). Progress curves of adenine released from 16 µM of hsDNA catalyzed by ricin at CPE in the acetate buffer pH 4.6 against the product of time with enzyme amount. The solid curve represents the linear fitting to the data. Table S1: ricin purification steps.
