# Supplementary material for: An Electrochemical Approach to Follow and Evaluate the Kinetic Catalysis of Ricin on hsDNA
Source: Life (Basel). 2021 Apr 29;11(5):405. doi: 10.3390/life11050405 (PMC8145570; doi:10.3390/life11050405)
Supplement: Supplementary file 1 [file life-11-00405-s001.zip › life-1145852-supplementary.pdf]

life

MDPI

Article

An Electrochemical Approach to Follow and Evaluate the Kinetic Catalysis of Ricin on hsDNA

George Oliveira * and José Maurício Schneedorf

Department of Biochemistry, Federal University of Alfenas, Alfenas 37130-000, Brazil; jose.dasilva@unifal-mg.edu.br
* Correspondence: george.oliveira@unifal-mg.edu.br

Abstract: International authorities classify the ricin toxin, present in castor seeds, as a potential agent for use in bioterrorism. Therefore, the detection, identification, and characterization of ricin are considered the first actions for its risk assessment during a suspected exposure, parallel to the development of therapeutic and medical countermeasures. In this study, we report the kinetic analysis of electro-oxidation of adenine released from hsDNA by the catalytic action of ricin by square wave voltammetry. The results suggest that ricin-mediated adenine release exhibited an unusual kinetic profile, with a progress curve controlled by the accumulation of the product and the values of the kinetic constants of 46.6 μM for *Km* and 2000 min^{-1} for *kcat*, leading to a catalytic efficiency of 7.1×10^5 s^{-1} M^{-1}.

Keywords: ricin; square wave voltammetry; kinetic analysis; hsDNA

Citation: Oliveira, G.; Schneedor, J.M. An Electrochemical Approach to Follow and Evaluate the Kinetic Catalysis of Ricin on hsDNA. *Life* **2021**, *11*, 405. https://doi.org/10.3390/life11050405

Academic Editor: Angela Tramonti and Roberto Contestabil

Received: 28 February 2021
Accepted: 19 April 2021
Published: 29 April 2021

Publisher's Note: MDPI stays neutral with regard to jurisdictional claims in published maps and institutional affiliations.

Copyright: © 2021 by the authors. Submitted for possible open access publication under the terms and conditions of the Creative Commons Attribution (CC BY) license (http://creativecommons.org/licenses/by/4.0/).

Supplementary Materials:

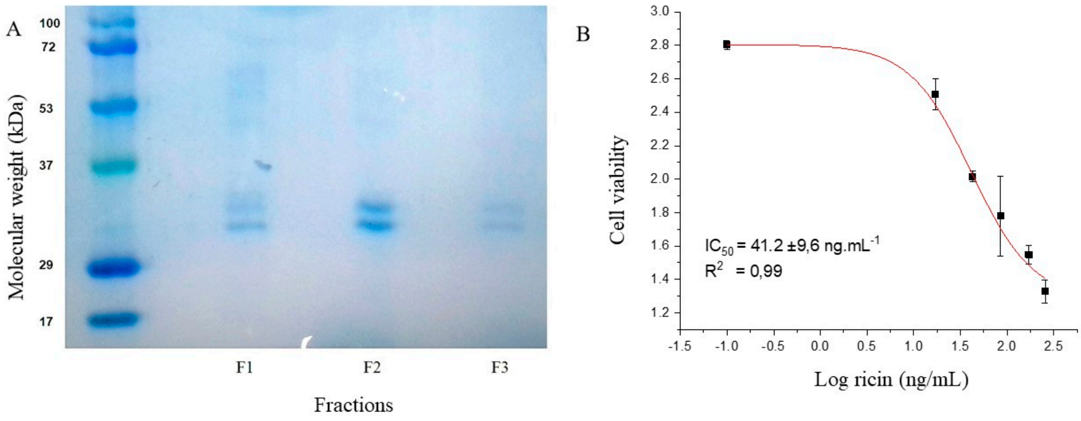

Figure S1. Purification and characterization of ricin. (A) SDS-PAGE (12 %) of samples under reduced conditions, (B) Effect of a pooled fraction of purified ricin on the viability of HEP2 cells for IC_{50} determination. The line represents a logistic equation fitted to the data.

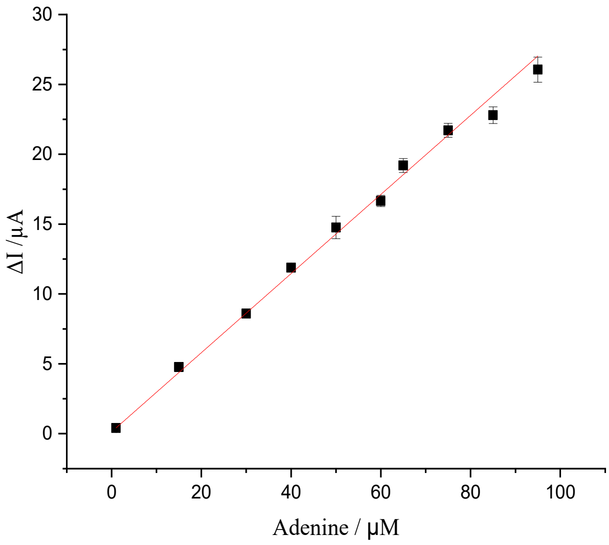

Figure S2. Analytical curve of adenine obtained by successive additions of standard adenine aliquots in a range from 0 to 95 µM in a solution containing hsDNA at 16 µM.

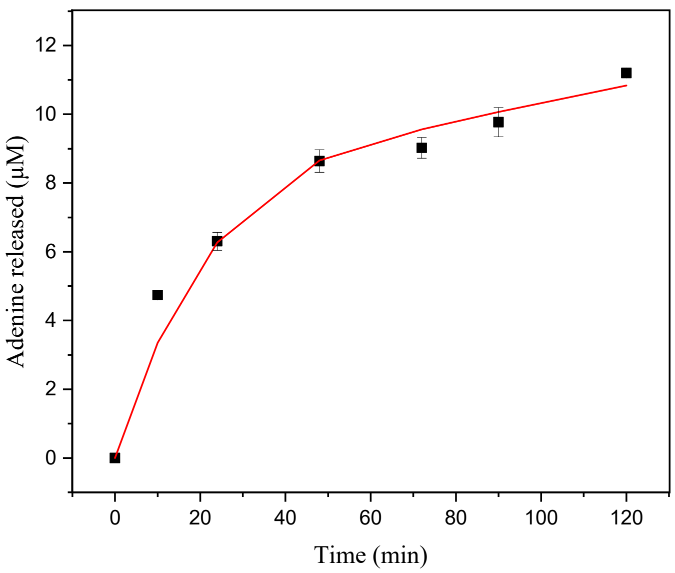

Figure S3. Progress curve of adenine release from hsDNA (5 µM) catalyzed by ricin at 1.5 nM as obtained by HPLC. The solid curve represents the fit of eq 1 to the experimental data.

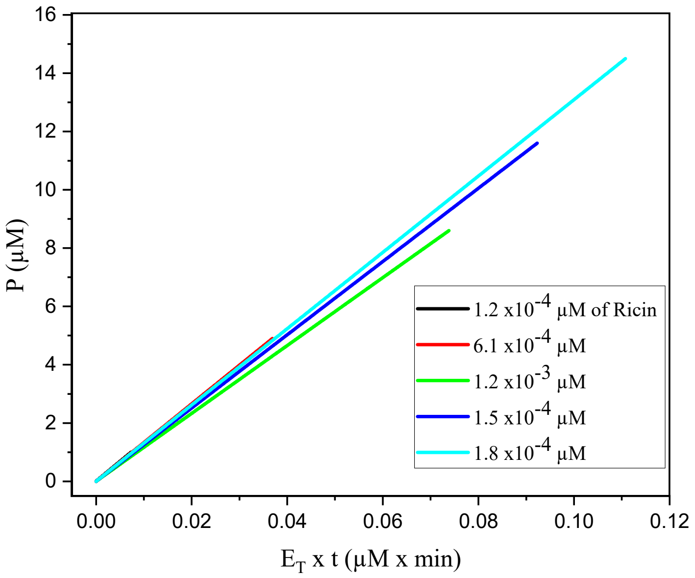

Figure S4. Selwyn's test (enzyme inactivation). Progress curves of adenine released from 16 µM of hsDNA catalyzed by ricin at CPE in the acetate buffer pH 4.6 against the product of time with enzyme amount. The solid curve represents the linear fitting to the data.

Table S1. Ricin purification steps.

Purification Stage	Total Proteins (μg)	Total Activity μM.min^{-1}	Specific Activity μM.min^{-1}. μg^{-1}	Purification Factor Times	Recovery (%)
Crude extract	1094.8	2.6	0.0024	1.0	100.0
Ammonium Sulfate (60%)	370.9	2.2	0.0059	2.4	82.6
Pre-purified extract (Sephadex G-50)	20.6	1.5	0.0726	30.0	56.6
Purified sample (Sephadex G-100)	4.6	1.3	0.2778	114.9	48.3
